# The suitability of panoramic radiographs for clinical decision making regarding root angulation compared to cone-beam computed tomography

**DOI:** 10.1186/s12880-021-00619-y

**Published:** 2021-05-24

**Authors:** Athbi Alqareer, Rania Nada, Aref Ghayyath, Mariam Baghdady, Veerasathpurush Allareddy

**Affiliations:** 1 Department of Developmental and Preventive Sciences, Faculty of Dentistry, Kuwait University, P.O. Box 24923, 13110 Safat, Kuwait; 2 Department of Diagnostic Sciences, Faculty of Dentistry, Kuwait University, Safat, Kuwait; 3 Department of Orthodontics, Brodie Craniofacial Endowed Chair, College of Dentistry, University of Illinois at Chicago, Chicago, USA

**Keywords:** Cone-beam CT, Panoramic radiograph, Root angulation, Bracket repositioning, Root proximity

## Abstract

**Background:**

The study compared clinical decisions regarding root angulation correction and root proximity based on the interpretation of Panoramic (PAN) versus Cone-Beam Computed Tomography (CBCT) images.

**Methods:**

A total of 864 teeth from 36 existing, radiographic patient records at a university dental clinic with concurrent PAN and CBCT images were assessed using PANs, then using CBCTs in a blinded manner by two orthodontists. Teeth were rated regarding the need for root repositioning, the direction of repositioning and existence of root proximity. Frequencies, rating time and intra- and inter-examiner Cohen’s Kappa were calculated.

**Results:**

There was 73.7–84.5% agreement between PAN-based and CBCT-based orthodontists’ decisions regarding the need to reposition roots. Root proximity was more frequently reported on PANs than CBCTs by one examiner (*p* = 0.001 and *p* = 0.168). Both PANs and CBCTs had moderate to substantial intra-examiner, within-radiograph-type reliability with Kappa values of 0.686–0.79 for PANs, and 0.661 for CBCTs (*p* < 0.001). Inter-examiner and inter-radiograph-type Kappa values ranged from 0.414 to 0.51 (*p* < 0.001). Using CBCT decisions as a reference, 78.9% of PAN decisions were coincident, 9.3% would have been repositioned on CBCT but not on PAN, 11.3% would not have been repositioned on CBCT but were on PAN, and 0.3% would have been repositioned in the opposite direction on CBCT versus PAN. Additionally, CBCT images required more time per tooth to assess than PANs (*p* < 0.001).

**Conclusions:**

PAN-based clinical decisions regarding root angulation had comparable statistical reliability and substantial agreement with CBCT-based clinical decisions.

## Background

During orthodontic treatment, alignment of the roots of the teeth in good axial angulation is critical for a good, stable orthodontic result [1]. Often, orthodontists assess root angulation and parallelism during the later stages of treatment using panoramic radiographs (PANs). This method for assessment is deemed acceptable and practical based on the examination guidelines provided by the American Board of Orthodontics [2]. Based on the evaluation of PANs, the orthodontist would often decide to re-bracket some teeth or place finishing bends if ideal root alignment was not met. PANs are also routinely used by orthodontists for other diagnostic purposes like identifying missing, impacted, or supernumerary teeth, assessing root length, and identifying gross deviations in the mandible and temporomandibular joints.

The accuracy of using PANs for checking root angulation has come into question in the literature. Several studies have demonstrated that panoramic imaging has limits when used for assessing the mesio-distal angulation of teeth [3, 4]. Such studies indicated the superior accuracy of using Cone-Beam Computed Tomography (CBCT) in determining root angulation. Despite the benefit of representing dentofacial structures in a 1:1 ratio due to its isotropic voxels, CBCTs expose patients to higher levels of radiation in comparison to PANs. A single CBCT scan using a field of view (FOV) necessary to assess the entire dentofacial area has an effective dose between 36 and 1073 µSv [5, 6]. This effective dose is higher than the effective dose of digital PAN (23 µSv) [7] and digital lateral cephalometric (4.5 µSv) [8] skull views. Coupled with the fact that the orthodontist may require multiple exposures over the course of treatment, this may deem CBCT use problematic, especially in children, in light of the As Low as Reasonably Achievable (ALARA) principle. CBCT images may also require more expertise and time to interpret when compared to PANs.

Most studies comparing CBCT and PAN imaging focused on determining the differences in degrees of root angulation and not necessarily on clinical decisions or bracket repositioning outcomes resulting from these differences. In practice, what is important is the qualitative decision of whether the orthodontist should reposition a bracket or not and in which direction. Although CBCT imaging may increase clinicians’ confidence in visualizing root angulation, it has yet to be determined whether the differences between PANs and CBCTs will result in clinically significant changes in decision making. The premise of the current study is that orthodontists do not normally measure angulation of roots in degrees but rather categorically assess the root for problems in angulation. If this is the usual way orthodontist assess roots, and if the clinical and visual judgment of the orthodontists is a limiting factor in the process, would the relatively inferior imaging modality of panoramic radiographs result in similar clinical decisions compared to CBCTs? Hence, the aim of the study was to compare clinical decisions regarding root angulation correction and root proximity based on the interpretation of PAN versus CBCT images. We hypothesized that clinical decisions differ based on the interpretation of PAN versus CBCT images.

## Materials and methods

### Institutional review board and ethical approval

The study utilized existing radiographic images that were already acquired as part of patients’ dental treatment. No additional radiographs were exposed for the present study. The study design was approved by the Institutional Review Board (IRB) of the Office of Human Subjects Research at Kuwait University. The IRB protocol # is VDR/EC/30.

### Images

The Kuwait University Dental Center imaging database (Romexis 4.4.2.R, Planmeca, Helsinki, Finland) was searched for patients with existing CBCT images with an FOV of 16 × 10 cm or higher and a concurrent PAN as part of their dental treatment. The clinical indications for specific radiographic exposures was not known to the authors. However, PANs are typically used in this clinical facility during initial dental screening. CBCT images may have been requested to evaluate the position of third molars, evaluate previously endodontically treated teeth, plan for dental implants and/or any other clinical indication during the course of patients’ treatment. Images with small FOV CBCT (less than 16 × 10 cm), craniofacial anomalies, six or more missing permanent teeth, PAN and CBCT exposures more than a month apart, patient positioning errors, and/or distinguishing features that could affect the blinding of examiners were excluded. The radiographic database had 1008 patient records with a CBCT image. Of these records, 966 were excluded due to a small FOV or lack of PAN image. The preliminary sample screened by the radiologists consisted of 42 patient records of which 6 were excluded for the presence of identifying features. The final sample included 36 cases.

The PANs were acquired using a Planmeca 3D Mid® (Helsinki, Finland) operated at a range of 64–72 kV, 6.3–12 mA and exposure times of 16–19 s as per clinical protocols and manufacturer’s recommendations. The CBCTs were also acquired using Planmeca 3D Mid® (Helsinki, Finland) operated at 90 kV, 12 mA and an average scanning time of 14 s also as per clinical protocols and manufacturer’s recommendations. Both image types were produced using the same sensor. However, it is important to emphasize that the PANs were actual panoramic exposures and not reconstructed from the CBCT volumes. The voxel size for CBCT images ranged from 200 to 400 µm. The effective doses for the radiographic equipment used in this study according to the literature ranged from 122 µS to 283 µSv for an FOV of 10 × 10 to 16 × 16 cm on CBCT [5, 6]. Our study used both 16 × 10 and 16 × 16 cm FOVs. The effective dose for PANs was 23 µSv [7].

### Radiographic assessment

Radiographic images were viewed using the Romexis® Software (version 4.4.2.R, Planmeca, Helsinki, Finland) on an HP ProDesk personal computer (Hewlett-Packard, Palo Alto, CA, USA) operated by a 64-bit Microsoft Windows® 8.1 Pro operating system (Microsoft Corporation, Redmond, Washington, USA) with 16 gigabytes of RAM and a 2 gigabyte AMD Radeon HD 5450 graphics card (Advanced Micro Devices, Santa Clara, CA, USA). The monitor used for viewing the images was an HP V242 backlit LED monitor with a native resolution of 1920 by 1080 pixels (Hewlett-Packard, Palo-Alto, CA, USA). The CBCT images were viewed in Digital Imaging and Communications in Medicine (DICOM) format in the three orthogonal planes (axial, coronal, and sagittal) that represented the primary reconstructed images provided by the manufacturer’s software. The examiners had the freedom to window and level the images. They also were able to rotate CBCT images when necessary to achieve a better angle of view. No additional multiplanar reconstructions were made (e.g. panoramic reconstruction). Both orthodontists received the basic training to use the software and they have around 6–11 years experience in treating orthodontic patients and viewing CBCT volumes.

Each radiographic image was anonymized and given a unique, blinded ID by the radiologists in the study prior to presentation to the orthodontists for evaluation. Two orthodontists evaluated each tooth on the PAN and CBCT images (first molar to first molar) independently. The radiographs were randomized and blinded so that the orthodontists were unable to correlate CBCTs with PANs. The orthodontists had a pilot session in which they went over the workflow of the image evaluation procedure. However, no attempts were made to force an agreement between the two assessors and ratings were totally independent. All PANs were rated first, then 3 weeks later all CBCTs were rated.

For each tooth, the orthodontist had to pick between three clinical decisions: (1) Do not adjust root position, (2) adjust root position to tip root distally (DRT), and (3) adjust root position to tip root mesially (MRT). The orthodontists also assessed the roots for the presence of dilaceration or root proximity (radiographic appearance of contact between roots). For each image, the time spent by the orthodontist evaluating the imaging modality was recorded. The criterion used to decide on whether or not to reposition a root was the clinical judgment of the orthodontist (i.e. the orthodontist had to behave as if the radiographic image was of an actual orthodontic patient in later stages of treatment in making his/her decisions).

Outcomes of interest: The primary outcome measure was the clinical decision to reposition the root or not and in which direction based on the PAN as compared to CBCT. Other variables tested included the number of repositions recommended per image, time needed for evaluation, and the reliability within and between imaging modalities and examiners.

### Statistical approach

The unit of analysis for computing the sample size was the individual evaluated tooth. Sample size estimation was done using the formula provided by Buderer since the study dealt with the accuracy of a diagnostic test [9]. The first step was to determine the “number free from disease” (i.e. False Positive “FP” + True Negative “TN”) using the formula ($FP+TN={Z}^{2}\frac{SN(1-SN)}{{W}^{2}}$ for sensitivity based calculation, and $FP+TN={Z}^{2}\frac{SP(1-SP)}{{W}^{2}}$ for specificity based calculation). Then N was calculated using the formula (${N}_{1}=\frac{FP+TN}{P}$ for Sensitivity based calculation, and${N}_{2}=\frac{FP+TN}{1-P}$). Where P (prevalence) was set to 0.15, W (accuracy) was set to 0.1, and Z was set to 1.96 based on a confidence interval of 95%. Using various assumptions of expected/desired sensitivities and specificities (SN and SP), the number of required teeth ranged between150 to 550. Choosing the 550 tooth estimates and assuming a minimum of 15–16 evaluated teeth per patient, the sample size planned was 35 patients. This sample size would achieve 80% or more power for Cohen’s Kappa tests according to calculations provided by Bujang and Baharum [10].

Chi-square test was used to test categorical variables while the Pearson correlation coefficients and paired-t tests were used to examine continuous outcomes between the PAN and CBCT images. The repeatability of decisions based on PANs and CBCTs were assessed by repeating the ratings on 10 randomly selected cases. These cases were rated twice using PANs and twice using CBCTs in a blinded manner two to three weeks apart. Cohen’s Kappa statistic was used to assess reliability within and between examiners and imaging modality types. A combined scheme for the interpretation of the Kappa values merging the guideline provided by Landis and Koch [11] and McHugh [12] where values of 0.4–0.6 were considered “weak to moderate”, 0.6–0.8 “moderate to substantial” was used. All statistical tests of associations were two-sided and a *p* value of < 0.05 was deemed to be statistically significant. Statistical analyses were conducted using IBM® SPSS® Statistics 25 software (Armonk, New York, USA).

## Results

Overall, 864 teeth were rated, of which 1 tooth was deemed not possible to rate by both examiners. Frequencies of repositioning decisions, missing and dilacerated teeth are summarized in Table 1. The total agreement percentages were calculated by adding the total number of agreement incidences (the diagonal cells) divided by the total number of rated teeth. There was 73.7 and 84.5% overall agreement in clinical decisions between PANs and CBCTs for examiner 1 and 2 respectively. As maybe expected, there was complete agreement regarding missing teeth. Overall, repositioning of roots was recommended for 25 and 24.2% of rated teeth based of PAN and CBCT images respectively for examiner 1. While for examiner 2, the frequencies were 14 and 11.2%.

**Table 1:** Frequencies of clinical decisions cross-tabulation comparing PAN to CBCT decisions

	CBCT decision				
PAN decision	Reposition	Don’t reposition	Missing	Dilacerated	Total
Examiner 1					
Reposition	103 (49.3%)	115 (18.7%)	0 (0%)	1 (20%)	219 (25%)
Don’t reposition	104 (49.8%)	497 (80.9%)	0 (0%)	3 (60%)	604 (70%)
Missing	0 (0%)	0 (0%)	35 (100%)	0 (0%)	35 (4.1%)
Dilacerated	2 (1%)	2 (0.3%)	0 (0%)	1 (20%)	5 (0.6%)
Total	209 (100%)	614 (100%)	35 (100%)	5 (100%)	863 (100%)
Examiner 2					
Reposition	47 (48.5%)	7 (10%)	0 (0%)	2 (22.2%)	121 (14%)
Don’t reposition	47 (48.5%)	642 (88.9%)	0 (0%)	2 (22.2%)	691 (80.1%)
Missing	0 (0%)	0 (0%)	35 (100%)	0 (0%)	35 (4.1%)
Dilacerated	3 (3.1%)	8 (1.1%)	0 (0%)	5 (55.6%)	16 (1.9%)
Total	97 (100%)	722 (100%)	35 (100%)	9 (100%)	863 (100%)

After excluding all missing and dilacerated teeth. The sensitivity and specificity was calculated for PAN-based decisions using CBCT-decisions as a gold standard. The sensitivity ranges between 49.3 to 50%; and, specificity between 81.3 and 89.9%.

### Root proximity

After excluding missing and dilacerated teeth, orthodontists were more likely to mark teeth as appearing to be in contact on the PANs than on CBCTs. The difference was statistically significant for examiner 1 (*p* = 0.001) and not significant for examiner 2 (*p* = 0.168) using Fisher’s exact test, Table 2. Based on CBCT decisions as a standard, PAN decisions regarding root proximity had a sensitivity of 20–29.4% and a specificity of 95.7–96.5%.

**Table 2:** Root proximity frequencies cross-tabulation comparing PAN to CBCT

	CBCT decision		
PAN decision	Proximity present	Proximity absent	Total
Examiner 1			
Proximity present	5 (29.4%)	34 (4.3%)	39 (4.8%)
Proximity absent	12 (70.6%)	756 (95.7%)	767 (95.2%)
Total	17 (100%)	790 (100%)	807 (100%)
Examiner 2			
Proximity present	1 (20%)	28 (3.5%)	29 (3.6%)
Proximity absent	4 (80%)	774 (96.5%)	778 (96.4%)
Total	5 (100%)	802 (100%)	807 (100%)

### Reliability analysis

A total of 216 teeth were evaluated for intra-examiner reliability within the same image type, while the whole sample (863 teeth) was used for inter-examiner reliability and intra-examiner reliability between PAN and CBCT images. The results of reliability analysis for intra- and inter-examiner and intra- and inter-imaging-modality type are shown in Table 3. Both PAN to PAN and CBCT to CBCT decisions had substantial reliability.

**Table 3:** Reliability of clinical decisions intra- and inter-examiner and intra- and inter-imaging-modality

	n	% agreement	Kappa	*p* value
Examiner 1 PAN to PAN	216	89.4%	0.790	< 0.001
Examiner 1 CBCT to CBCT	216	84.7%	0.661	< 0.001
Examiner 1 PAN to CBCT	863	73.2%	0.424	< 0.001
Examiner 2 PAN to PAN	216	90.7%	0.686	< 0.001
Examiner 2 CBCT to CBCT	216	89.8%	0.661	< 0.001
Examiner 2 PAN to CBCT	863	84.2%	0.510	< 0.001
Examiner 1 PAN to Examiner 2 PAN	863	76.1%	0.433	< 0.001
Examiner 1 CBCT to Examiner 2 CBCT	863	77.2%	0.414	< 0.001

#### Clinical decision comparison between PAN and CBCT images

After excluding teeth marked as missing or dilacerated (n = 807), PAN and CBCT decisions were compared regarding agreement for each examiner. These values are presented for the overall sample and stratified by tooth type in Table 4. The highest agreement was noted at mandibular molars for both examiners while the least agreement was observed at maxillary laterals for examiner 1 and maxillary canines for examiner 2.

**Table 4:** Clinical decision agreement between PAN and CBCT by tooth type

Tooth type	n	Examiner 1 % agreement	Examiner 2 % agreement
Mx. molars	64	76.6	85.9
Mx. premolars	125	65.6	84
Mx. canines	69	87	66.7
Mx. laterals	71	60.6	78.9
Mx. centrals	72	81.9	91.7
Md. molars	67	83.6	98.5
Md. premolars	133	72.2	85
Md. canines	67	71.6	83.6
Md. laterals	69	60.9	81.2
Md. centrals	70	74.3	97.1
Total	807	72.7	85.1

**Fig. 1:**
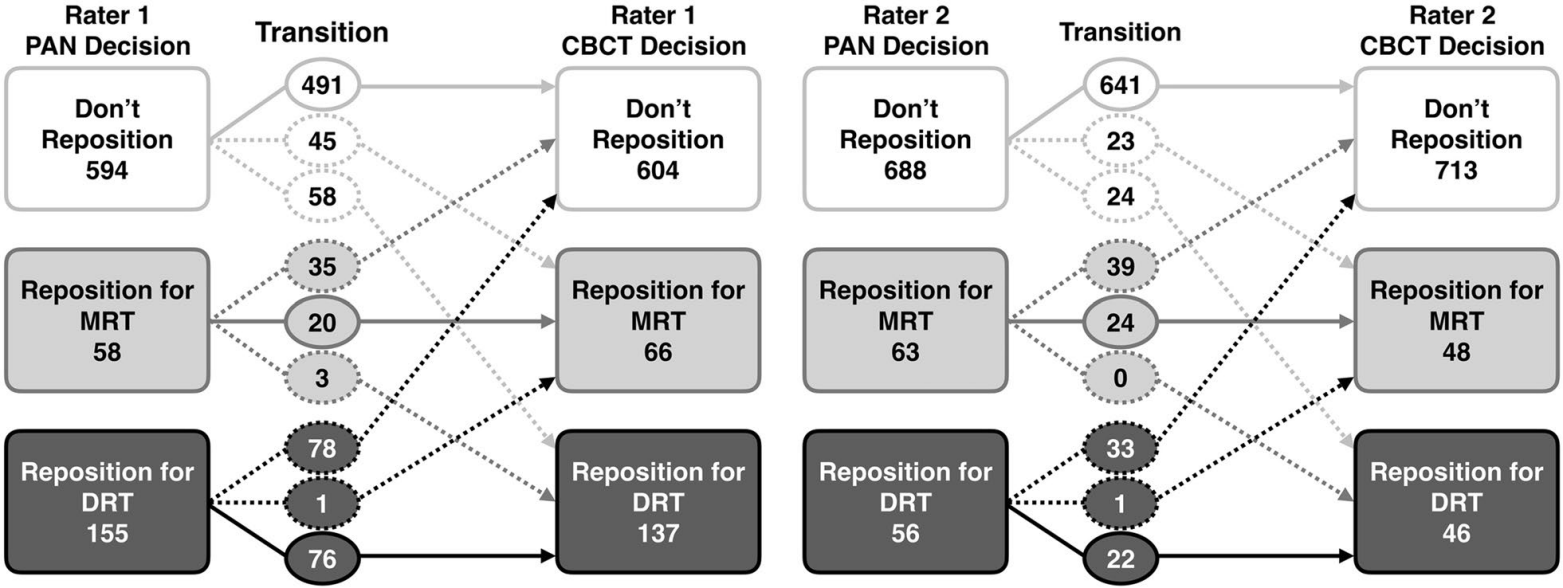
Clinical decision flowchart comparing PAN to CBCT decisions. Solid arrows indicate agreement between PAN-based and CBCT-based clinical repositioning decisions. Dashed arrows indicate disagreement between PAN-based and CBCT-based clinical repositioning decisions. MRT, mesial root tip; DRT, distal root tip

**Fig. 2:**
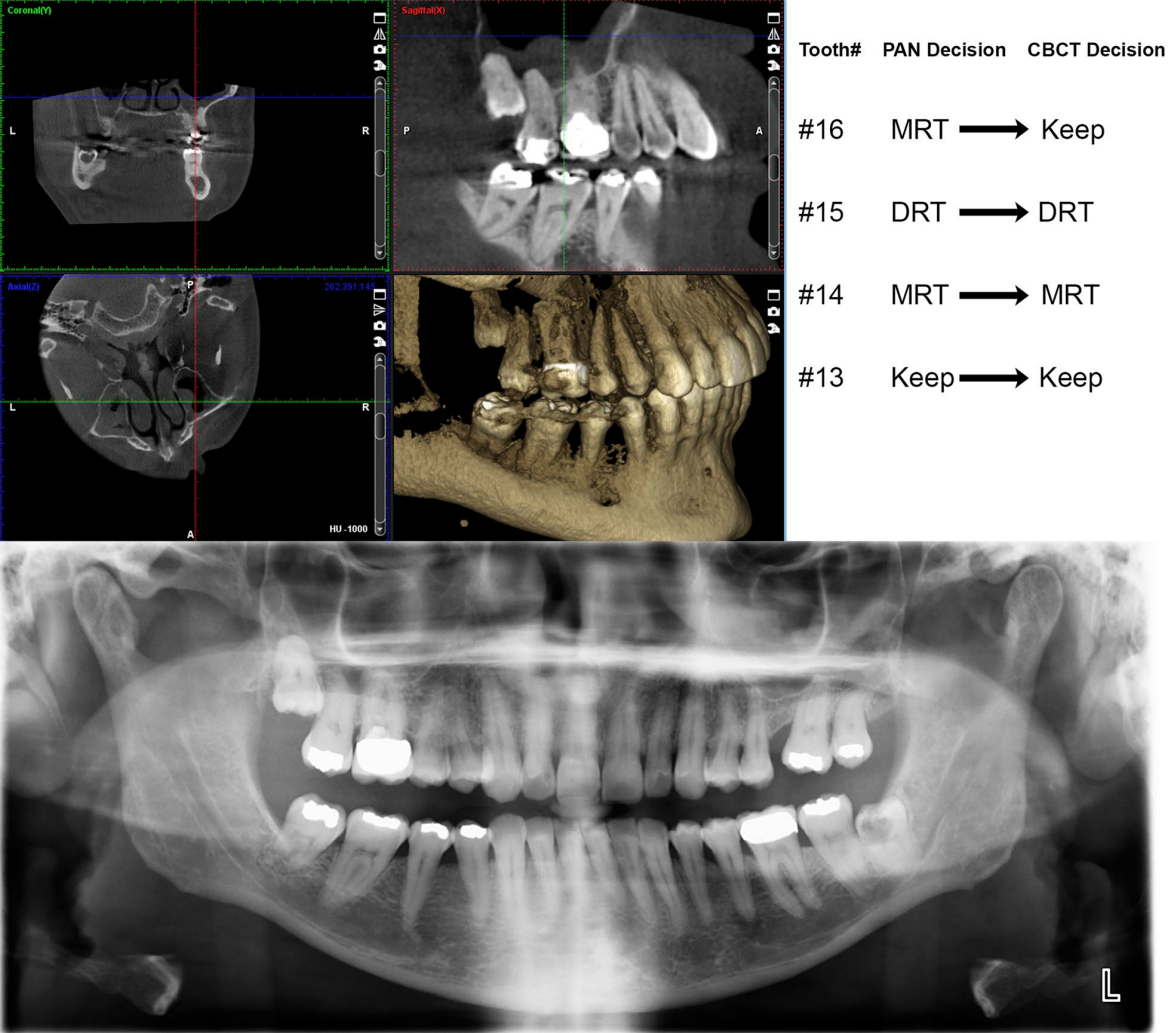
An example of repositioning decisions made by examiner 1 for the upper right quadrant of displayed CBCT and PAN images are shown on the upper right portion of the figure. The CBCT view displaying right side teeth with focus on the upper right quadrant is shown on the top left side of the figure. The corresponding PAN image is displayed on the bottom of the figure

The changes in clinical decisions, including root repositioning direction, made by each orthodontist were compared between PANs and CBCTs and displayed as a flowchart in Fig. 1. Using CBCT-based decisions as a reference, PAN decisions were further classified into four categories: (1) coinciding with CBCT, (2) would have been repositioned based on CBCT but was not based of PAN, (3) would have not been repositioned on CBCT but was on PAN (4) would have been repositioned in the opposite direction based on CBCT compared to PAN. When the findings of the two examiners were averaged, 78.9% of the PAN decisions were classified as coincident (1), 9.3% as “would have been repositioned” (2), 11.3% as “would not have been repositioned” (3), and only 0.3% as “opposite direction” (4). A representative, visual case example comparing PAN and CBCT-based decisions for examiner 1 for the upper right quadrant of the mouth can be seen in Fig. 2 with the corresponding images.

#### Rating time

There was no significant correlation between the number of teeth requiring repositions on the image and the time needed to rate the image (r = 0.166, *p* = 0.165, Pearson correlation). To adjust for missing teeth, the time spent by each examiner rating each image was divided by the number of present teeth. This outcome variable (time-per-tooth) was then used to compare the two radiographic modalities. Since the outcome variable was normally distributed according to the Kolmogorov–Smirnov test (*p* = 0.053) and since the observations were related, the paired t-test was used to test for statistical differences. The difference in time-per-tooth was statistically significantly different between the two types of images (*p* < 0.001) with the average time-per-tooth of 9.12 ± 2.64 s for CBCT and 4.98 ± 2.94 s for PAN. On average CBCT tooth ratings required 82% more time than PAN ratings.

## Discussion

The purpose of the present study was to determine differences between clinical decisions made based on PANs compared to CBCTs for the evaluation of the mesio-distal root angulation. CBCTs and PANs of 36 patients (a total of 864 teeth), were examined by 2 orthodontists. For each tooth, the orthodontist had to decide whether the tooth had a correct angulation or required either mesial or distal root tipping. The clinical decisions made based on PANs were then compared to the decisions made based on CBCTs.

Root angulation measurements on CBCT scans are considered more accurate than PANs as previously validated by Peck et al. [3]. However, the differences between the two radiographic projections were mostly evaluated in other studies from a geometric perspective based on angular values and a presumed clinically significant threshold set between 2.5° to 5° [4, 13]. The conclusion of previously mentioned studies was in agreement with that of a similar study by Owens and Johal [14] which compared the actual mesio-distal angulation of teeth measured on a typodont compared to that measured on PANs of the same typodont. Similar to Peck et al. [3], the greatest variation was found in the maxillary arch in the canine premolar area where the roots were projected as being more divergent, while in the mandibular arch the greatest variation was measured at the lateral incisor-canine region where the roots were projected as being more convergent. They concluded that this could lead the clinician to perform unnecessary or exaggerated root angulations. These studies however, did not allude to whether the observed differences resulted in a significant change in clinical outcomes, i.e. whether to reposition a bracket or place a finishing bend to adjust a tooth’s angulation or not.

The results of the present study showed that when it came to actual clinical decisions rather than angular measurements, there was a 72–85% agreement between clinical decisions made based on PANs and CBCTs. When it came to unnecessary root repositions, only an average of 11.5% of the clinical decisions to reposition made on PANs would have not been repositioned based of CBCT. This suggest that even though CBCT offered the clinician more accurate root angulation measurements when compared to PANs, this may have had little effect on actual clinical decisions in the current study. Perhaps clinicians might be aware of the limitations of panoramic projections and are able to account for them when making clinical decisions. Or perhaps there is some leeway in the degree of angulation a clinician is willing to accept.

In general, choosing an imaging modality should only be done after a thorough clinical examination indicates that more information is needed. Diagnostic radiology should be part of a larger system whose goal is to treat patients effectively and efficiently [15]. Although CBCT images may provide superior geometric accuracy when compared to PAN images, CBCT imaging has its drawbacks. The effective dose of CBCT using a medium to large FOV is significantly higher than PANs. This is especially important given the age group of orthodontic patients and if multiple scans will be required throughout treatment [5]. In practice, the orthodontist acquiring the CBCT scan is responsible for evaluating the entire volume not only the area of interest. In this study, the orthodontists were only required to evaluate root angulation on the acquired images yet CBCT assessment required 82% more time to rate than PANs. Evaluation time should also be another factor to consider when comparing the two radiographic modalities from a clinical perspective. Most orthodontist would likely have a higher familiarity with PANs than CBCTs.

A particular strength of the present study is that it compared two commonly used imaging modality in the most clinically relevant and practical way, namely, categorical decisions. This is more clinically applicable than measuring degrees of angulation of roots in geometric terms since such measurements are hardly ever performed by orthodontists in the clinic. Another design advantage of the study is that it did no include patients under active orthodontic treatment. This reduces the risk for changes in root position between the time the PAN and CBCT images were exposed. The findings of the present study suggests that perhaps when it comes to decisions to reposition teeth due to mesio-distal root angulation, the additional accuracy of CBCT did not offer a major advantage. One may speculate that because the clinicians’ judgment may be a limiting factor in such situations, the additional accuracy in terms of angular degrees did not translate into meaningful differences in clinical decision. In the clinic, root-repositioning decisions should always be based on a combination of radiographic findings and clinical assessment of teeth.

## Limitations

The study has several limitations and the findings should be interpreted keeping the limitations in perspective. The radiographic records used were not of orthodontic patients, which would have more closely represented the clinical situation in question. Because of the ethical challenges associated with the radiation exposure of CBCT to children and young adults, CBCT images of actual orthodontic patients were limited. A prospective approach to such a study design where patient have both PAN and CBCT taken would be unethical since patients would be unnecessarily exposed to a redundant radiographic image. Retrospectively this was possible; because, for most of the study subjects, the CBCT was requested as part the dental treatment of the patient to gain more information than the existing PAN provided. Arguably, with actual orthodontic patients, the presence of fixed appliances on the images could be speculated to further improve repositioning decisions by providing an additional visual orientation reference for the orthodontist. As stated earlier in the discussion, one could also argue that not including active orthodontic patients may be considered an advantage.

Another possible limitation of this study is that ratings were only done by two examiners. It is possible that differences in decision making would exist within the general population of orthodontists which may limit the generalizability of the study. However, the similarities in rating patterns between the two examiners, despite the lack of forced agreement, might indicate a trend for agreement between orthodontists in general. It is also worth mentioning that all the CBCT and PAN images were acquired using the same machine; hence, the findings of this study may not be readily applicable to other machines.

In this study, the clinical decisions on whether to correct root angulation based on CBCT images were used as a reference to judge PAN decisions, this is in itself an assumption as CBCT-based decisions are prone to errors as well. The observed “errors” in this study may be in part due to intra-examiner variability in decision-making. Even when CBCT decisions were repeated, some differences existed. In fact, the Kappa values for inter-PAN ratings were slightly higher than inter-CBCT ratings. In other words, these “errors” or disagreements could be at least partially due to the less than perfect reliability of decisions made based on either modality and not purely the result of inaccuracy of PAN decisions. Finally, the unit of analysis was the individual tooth. There is a likelihood of clustering effects of outcomes within patients.

## Conclusions

PAN-based clinical decisions regarding the need to orthodontically reposition roots due to mesio-distal angulation had comparable reliability and substantial agreement with CBCT-based clinical decisions.

## Data Availability

The datasets generated and/or analyzed during the current study are not publicly available due to limitations of ethical approval involving the patient data and anonymity but are available from the corresponding author on reasonable request.

## Abbreviations

ALARA: As low as reasonably achievable
CBCT: Cone beam computed tomography
DICOM: Digital imaging and communications in medicine
DRT: Distal root tip
FOV: Field of view
FP: False positive
IRB: Institutional review board
MRT: Mesial root tip
PAN: Panoramic radiograph
TN: True negative

## References

[1] Andrews LF (1972). The six keys to normal occlusion. Am J Orthod.

[2] Orthodontics TABo. Grading System for Dental Casts and Panoramic Radiographs. 2012; https://www.americanboardortho.com/media/1191/grading-system-casts-radiographs.pdf. 10.1016/s0889-5406(98)70179-9 9810056

[3] Peck JL, Sameshima GT, Miller A, Worth P, Hatcher DC (2007). Mesiodistal root angulation using panoramic and cone beam CT. Angle Orthod.

[4] Bouwens DG, Cevidanes L, Ludlow JB, Phillips C (2011). Comparison of mesiodistal root angulation with posttreatment panoramic radiographs and cone-beam computed tomography. Am J Orthod Dentofacial Orthop.

[5] Ludlow JB, Timothy R, Walker C (2015). Effective dose of dental CBCT-a meta analysis of published data and additional data for nine CBCT units. Dentomaxillofac Radiol.

[6] Pauwels R, Beinsberger J, Collaert B (2012). Effective dose range for dental cone beam computed tomography scanners. Eur J Radiol.

[7] Gavala S, Donta C, Tsiklakis K, Boziari A, Kamenopoulou V, Stamatakis HC (2009). Radiation dose reduction in direct digital panoramic radiography. Eur J Radiol.

[8] Grunheid T, Kolbeck Schieck JR, Pliska BT, Ahmad M, Larson BE (2012). Dosimetry of a cone-beam computed tomography machine compared with a digital x-ray machine in orthodontic imaging. Am J Orthod Dentofacial Orthop.

[9] Buderer NM (1996). Statistical methodology: I. Incorporating the prevalence of disease into the sample size calculation for sensitivity and specificity. Acad Emerg Med.

[10] Bujang MA, Baharum N (2017). Guidelines of the minimum sample size requirements for Kappa agreement test. Epidemiol Biostat Public Health.

[11] Landis JR, Koch GG (1977). The measurement of observer agreement for categorical data. Biometrics.

[12] McHugh ML (2012). Interrater reliability: the kappa statistic. Biochem Med (Zagreb).

[13] McKee IW, Williamson PC, Lam EW, Heo G, Glover KE, Major PW (2002). The accuracy of 4 panoramic units in the projection of mesiodistal tooth angulations. Am J Orthod Dentofacial Orthop.

[14] Owens AM, Johal A (2008). Near-end of treatment panoramic radiograph in the assessment of mesiodistal root angulation. Angle Orthod.

[15] Fryback DG, Thornbury JR (1991). The efficacy of diagnostic imaging. Med Decis Making.

